# Carriage of *Streptococcus pneumoniae*, *Haemophilus influenzae*, *Moraxella catarrhalis*, and *Staphylococcus aureus* in Indonesian children: A cross-sectional study

**DOI:** 10.1371/journal.pone.0195098

**Published:** 2018-04-12

**Authors:** Eileen M. Dunne, Chrysanti Murad, Sunaryati Sudigdoadi, Eddy Fadlyana, Rodman Tarigan, Sang Ayu Kompiyang Indriyani, Casey L. Pell, Emma Watts, Catherine Satzke, Jason Hinds, Nurhandini Eka Dewi, Finny Fitry Yani, Kusnandi Rusmil, E. Kim Mulholland, Cissy Kartasasmita

**Affiliations:** 1 Pneumococcal Research, Murdoch Children’s Research Institute, Parkville, Victoria, Australia; 2 Department of Paediatrics, The University of Melbourne, Parkville, Victoria, Australia; 3 Department of Biomedical Sciences, Division of Microbiology, Faculty of Medicine, Universitas Padjadjaran, Bandung, West Java, Indonesia; 4 Department of Child Health, Universitas Padjadjaran/Hasan Sadikin General Hospital, Bandung, West Java, Indonesia; 5 West Nusa Tenggara Province General Hospital, Mataram, West Nusa Tenggara, Indonesia; 6 Department of Microbiology and Immunology, The University of Melbourne at the Peter Doherty Institute for Infection and Immunity, Parkville, Victoria, Australia; 7 Institute for Infection and Immunity, St. George’s University of London, London, United Kingdom; 8 BUGS Bioscience, London Bioscience Innovation Centre, London, United Kingdom; 9 District Health Office of Central Lombok, Praya, West Nusa Tenggara, Indonesia; 10 Department of Child Health, Universitas Andalas, Padang, West Sumatra, Indonesia; 11 Department of Infectious Disease Epidemiology, London School of Hygiene and Tropical Medicine, London, United Kingdom; Universidad Nacional de la Plata, ARGENTINA

## Abstract

*Streptococcus pneumoniae* is an important cause of infection and commonly colonizes the nasopharynx of young children, along with other potentially pathogenic bacteria. The objectives of this study were to estimate the carriage prevalence of *S*. *pneumoniae*, *Haemophilus influenzae*, *Moraxella catarrhalis*, and *Staphylococcus aureus* in young children in Indonesia, and to examine interactions between these bacterial species. 302 healthy children aged 12–24 months were enrolled in community health centers in the Bandung, Central Lombok, and Padang regions. Nasopharyngeal swabs were collected and stored according to World Health Organization recommendations, and bacterial species detected by qPCR. Pneumococcal serotyping was conducted by microarray and latex agglutination/Quellung. Overall carriage prevalence was 49.5% for *S*. *pneumoniae*, 27.5% for *H*. *influenzae*, 42.7% for *M*. *catarrhalis*, and 7.3% for *S*. *aureus*. Prevalence of *M*. *catarrhalis* and *S*. *pneumoniae*, as well as pneumococcal serotype distribution, varied by region. Positive associations were observed for *S*. *pneumoniae* and *M*. *catarrhalis* (OR 3.07 [95%CI 1.91–4.94]), and *H*. *influenzae* and *M*. *catarrhalis* (OR 2.34 [95%CI 1.40–3.91]), and a negative association was found between *M*. *catarrhalis* and *S*. *aureus* (OR 0.06 [95%CI 0.01–0.43]). Densities of *S*. *pneumoniae*, *H*. *influenzae*, and *M*. *catarrhalis* were positively correlated when two of these species were present. Prior to pneumococcal vaccine introduction, pneumococcal carriage prevalence and serotype distribution varies among children living in different regions of Indonesia. Positive associations in both carriage and density identified among *S*. *pneumoniae*, *H*. *influenzae*, and *M*. *catarrhalis* suggest a synergistic relationship among these species with potential clinical implications.

## Introduction

*Streptococcus pneumoniae* (pneumococcus) is a Gram-positive bacterium that causes a wide range of infections including otitis media, pneumonia, sepsis, and meningitis. Pneumococcal disease is a major cause of pediatric morbidity and mortality worldwide, with disease primarily occurring in low and middle income countries [1]. The primary reservoir for pneumococci is the human nasopharynx, and nasopharyngeal carriage rates are highest among young children, ranging from 19–86% in different epidemiological settings [2]. Colonization of the nasopharynx is typically asymptomatic although can also be associated with mild respiratory symptoms such as runny nose [3]. Importantly, carriage is considered a prerequisite to pneumococcal disease and serves as the source of pneumococcal transmission [4]. High pneumococcal carriage density has been linked to respiratory infection and pneumonia in children, and implicated in transmission in animal studies [5, 6].

The nasopharynx is a complex and dynamic environment, where pneumococci interact with the host immune system and other colonizing bacteria, including potential pathogens such as *Haemophilus influenzae*, *Moraxella catarrhalis*, and *Staphylococcus aureus*. In some cases, these interactions are mutually beneficial, whereas in others there is evidence for competition [7]. A negative association between *S*. *pneumoniae* and *S*. *aureus* in children has been well-documented [8, 9]. In contrast, positive associations have been identified among *S*. *pneumoniae*, *H*. *influenzae* and *M*. *catarrhalis* [7]. Densities of *S*. *pneumoniae and* non-typeable *H*. *influenzae* (NTHi) are positively associated during co-colonization [10, 11].

Indonesia is a lower-middle income country consisting of over 10,000 islands and has the fourth highest population in the world, estimated at over 260 million (www.worldbank.org). Pentavalent vaccine, which protects against *H*. *influenzae* type B (Hib), was introduced in Indonesia in 2013. Pneumococcal conjugate vaccines are not part of the national immunization program, although regional introduction commenced in late 2017. Previous studies conducted in Indonesia have reported a 43–48% pneumococcal carriage prevalence among healthy young children, with serotypes 6A/B, 15B/C, 11A, 19F, and 23F most commonly identified [12–14]. In Lombok, a *H*. *influenzae* carriage rate of 32% was found in children under two years old prior to Hib vaccine introduction [15]. There are no published data on carriage of *S*. *aureus* or *M*. *catarrhalis* in healthy children in Indonesia. The objectives of the current study were to investigate the carriage of *S*. *pneumoniae*, *H*. *influenzae*, *M*. *catarrhalis*, and *S*. *aureus* in healthy young children in three diverse regions of Indonesia, and to examine potential interactions between these bacterial species. These aims were achieved using a cross-sectional study design.

## Methods

### Study design and participants

This cross-sectional study was conducted between February-March 2016 in six health centers (puskesmas) located in three regions of Indonesia: Bandung, West Java (Puskesmas Puter, Bandung city; Puskesmas Jaya Mekar, Padalarang district of Bandung); Central Lombok, West Nusa Tenggara (Puskesmas Praya, Praya city; Puskesmas Ubung, Central Lombok); and Padang, West Sumatra (Puskesmas Padang Timur in Padang city; Puskesmas Bungus, outside Padang city). A sample size of 100 participants per study region was selected based on an estimated pneumococcal carriage prevalence of 50% at 12–23 months of age [13]. This sample size would provide a precision (estimated by widths of 95% confidence intervals) for the prevalence rate of approximately 10% for each study region and 6% overall.

Health center staff identified and invited eligible children, with their parent or guardian, to the health center on designated study days. Written informed consent was obtained from the parents/guardians prior to the conduct of any study procedures. Study protocols and consent forms were approved by the Health Research Ethics Committee, Universitas Padjadaran Faculty of Medicine (Indonesia) and the Royal Children’s Hospital Human Research Ethics Committee (Australia; RCH HREC reference number 35258). Inclusion criteria were being 12–24 (inclusive) months of age and residing within the health center jurisdiction. Exclusion criteria were moderate or severe acute illness, temperature ≥ 38 °C, antibiotic use within the previous 14 days, or previous receipt of pneumococcal conjugate vaccine.

### Study procedures and laboratory analyses

Data on demographic characteristics, living conditions, and medical history were recorded on a case report form. A nasopharyngeal swab was collected and stored according to World Health Organization recommendations using a flocked, nylon swab [16]. Swabs were placed into 1 ml skim milk tryptone glucose glycerol media (STGG), stored in a cool box, and transported to the local laboratory within 6 hours of collection. Samples were then vortexed, aliquoted, and stored at ultra-low temperature (ULT). Samples from the Padang and Central Lombok sites were shipped to the central laboratory (Microbiology Laboratory, Faculty of Medicine, Universitas Padjadaran, Bandung) on dry ice and stored at ULT until analysis. Storage in STGG at -70°C is optimal for recovery of *S*. *pneumoniae* from nasopharyngeal samples [17].

#### STGG DNA extraction and qPCR

For DNA extraction, 200 μl of the STGG sample was thawed and bacteria pelleted by centrifugation at 5,500 *x g* for 8 min. Bacterial lysis was conducted by a 60 min incubation at 37°C in a 20 mM Tris-HCl, 2 mM sodium EDTA buffer containing 20 mg/ml lysozyme, 1% (v/v) Triton X-100, 0.075 mg/ml mutanolysin and 2 mg/ml RNase A, followed by the addition of proteinase K and Buffer AL from the QIAamp 96 DNA QIAcube HT Kit (Qiagen) and 30 min incubation at 56°C. Lysates were transferred onto the QIAcube HT instrument (Qiagen) and DNA extraction performed according to the manufacturer’s instructions. Pneumococci were detected using real-time quantitative PCR (qPCR) targeting the *lytA* gene [18]. qPCR was conducted in 25 μl reactions containing 5 μl of template DNA on an Applied Biosystems 7500 real-time PCR machine using TaqMan GeneExpression Mastermix (Applied Biosystems). *H*. *influenzae*, *M*. *catarrhalis*, and *S*. *aureus* were detected using the FTD Bacterial Pneumonia CAP qPCR kit (Fast-Track Diagnostics). Standard curves for quantification were prepared using plasmid standards containing a single copy of the target gene (Fast-Track Diagnostics). Results are reported as genome equivalents/ml (GE/ml), which approximates bacterial density assuming each bacterial cell contains one genome with a single copy of the target gene. For *H*. *influenzae*, *M*. *catarrhalis*, and *S*. *aureus*, samples with a Ct value < 35 were considered positive and 35–40 considered negative. A Hib-specific qPCR targeting the *bscB* gene was conducted on *H*. *influenzae*-positive samples [19]. For pneumococcus, samples with a Ct value < 35 were considered positive, and those from 35–40 considered equivocal and confirmed by culture. Limits of detection were 40 GE/ml for *S*. *pneumoniae*, 3,890 GE/ml for *H*. *influenzae*, 8,700 GE/ml for *M*. *catarrhalis*, and 3,570 GE/ml for *S*. *aureus*.

#### Culture and pneumococcal serotyping

Pneumococcal positive and equivocal samples were cultured overnight at 37°C in 5% CO_2_, on sheep or horse blood agar containing 5 μg/ml gentamicin. For all samples containing α-hemolytic growth, a representative colony was subcultured on non-selective blood agar with an optochin disk, and if optochin susceptible, stored in STGG at ULT. The remaining growth from the original culture plate was emulsified into 1 ml phosphate-buffer saline, bacteria pelleted by centrifugation at 11,300 *x g* for 5 min, and stored at ULT. DNA was extracted from culture pellets for microarray as described above, without the addition of mutanolysin. Molecular serotyping by microarray was conducted using Senti-SP v1.6 (BUGS Bioscience) as previously described [20]. Stored isolates were serotyped by latex agglutination as previously described [21, 22]. If a serotype could not be determined by latex agglutination, Quellung was conducted using commercially available pneumococcal antisera (Statens Serum Institut, Copenhagen, Denmark) [23]. Latex/Quellung results were used for samples with growth of only one alpha-hemolytic colony or if DNA concentrations were too low to determine serotype by microarray. Serotyping results were consistent between the two methods unless otherwise noted. Serotypes 15B and 15C were reported as 15B/C as this serotype is known to interconvert [24].

#### Statistical analysis

Statistical analyses were conducted using Stata version 14.2 (StataCorp LLC) and GraphPad Prism version 7.03 for Windows (GraphPad Software). The chi-squared test was used to analyze categorical data. Continuous data were assessed for normality and the t-test or Mann-Whitney test were used to compare two groups and ANOVA or Kruskall-Wallis for multiple groups, as appropriate. Bacterial density data were log_10_ transformed prior to analysis. Logistic regression models were used to examine differences in carriage prevalence among sites and relationships between bacterial species. Potential risk factors shown in Table 1 were tested by univariable analysis and those found to be significantly associated with odds of carriage were included in a multivariable model to calculate adjusted odds ratios. Ethnicity was not included in the model due to co-linearity with site. P < 0.05 was considered significant. Spearman’s correlation was used to examine the relationship between densities of bacterial species.

**Table 1:** Characteristics of study participants.

Characteristics	Bandung region (total = 100) N (%)	Central Lombok (total = 101) N (%)	Padang region (total = 101) N (%)	P value^a^
Sex				
Male	42 (42.0)	50 (49.5)	52 (51.5)	0.365
Female	58 (58.0)	51 (50.5)	49 (48.5)	
Age (months)				
Mean ± SD	19.3 ± 2.8	18.8 ± 3.4	18.5 ± 3.6	0.198
Weight (kg)				
Mean ± SD	10.0 ± 1.4	9.2 ± 1.1	9.3 ± 1.4	<0.0001
Height (cm)				
Mean ± SD	77.7 ± 3.3	77.9 ± 4.0	80.7 ± 4.7	<0.0001
Ethnicity^b^				
Sundanese	91 (91.0)	0 (0.0)	0 (0.0)	<0.001
Sasak	0 (0.0)	99 (98.0)	0 (0.0)	
Minangkabau	0 (0.0)	0 (0.0)	97 (96.0)	
Other	9 (9.0)	2 (2.0)	4 (4.0)	
Residence				
Urban	50 (50.0)	51 (50.5)	51 (50.5)	0.997
Semi-rural	50 (50.0)	50 (49.5)	50 (49.5)	
Paternal education				
None	0 (0.0)	7 (6.9)	3 (3.0)	<0.001
Elementary school	6 (6.0)	19 (18.8)	7 (6.9)	
Junior high school	21 (21.0)	15 (14.8)	25 (24.8)	
Senior high school	50 (50.0)	44 (43.6)	61 (60.4)	
University	23 (23.0)	16 (15.8)	5 (5.0)	
Maternal education				
None	0 (0.0)	8 (7.9)	3 (3.0)	<0.001
Elementary school	9 (9.0)	15 (14.8)	6 (5.9)	
Junior high school	27 (27.0)	31 (30.7)	20 (19.8)	
Senior high school	45 (45.0)	39 (38.6)	63 (62.4)	
University	19 (19.0)	8 (7.9)	9 (8.9)	
Parental monthly income^c^				
Declined to answer	2 (2.0)	0 (0.0)	0 (0.0)	0.044
< 500,000 IDR	0 (0.0)	31 (30.7)	12 (11.9)	
500,000 IDR—Regional minimum salary	57 (58.2)	38 (37.6)	72 (71.3)	
> Regional minimum salary	41 (41.8)	32 (31.7)	17 (16.8)	
Number of children < 5y in the household				
1	77 (77.0)	88 (87.1)	75 (74.3)	0.362
2	18 (18.0)	11 (10.9)	19 (18.8)	
3	4 (4.0)	2 (2.0)	6 (5.9)	
4	1 (1.0)	0 (0.0)	1 (1.0)	
URTI symptoms^d^				
No	69 (69.0)	92 (91.1)	79 (78.2)	0.001
Yes	31 (31.0)	9 (8.9)	22 (21.8)	

^a^Chi-squared test for categorical data; t-test for continuous data

^b^As reported by parent/guardian

^c^IDR = Indonesian rupiah; Regional minimum salary rates (2016) were 1,800,725 IDR in Padang, 2,626,940 IDR in Bandung, and 1,550,000 IDR in Lombok

^d^Upper respiratory tract infection (URTI) symptoms include rhinorrhea, cough, and/or tonsillitis

## Results

**Fig 1:**
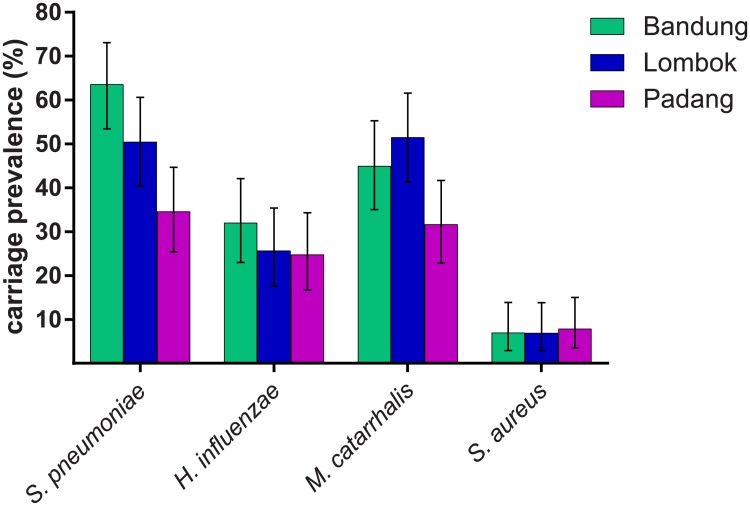
Nasopharyngeal carriage prevalence (%) of *S*. *pneumoniae*, *H*. *influenzae*, *M*. *catarrhalis*, and *S*. *aureus* in Indonesian children aged 12–24 months. Results are shown by region (Bandung, Padang, and Lombok). Error bars represent 95%CI.

A total of 302 children were included in this study, with participant characteristics shown in Table 1. Overall, pneumococcal carriage prevalence was 49.5% (95%CI 43.7–55.3), followed by 42.7% (95%CI 37.1–48.5) for *M*. *catarrhalis*, 27.5% (95%CI 22.5–32.9) *for H*. *influenzae*, and 7.3% (95%CI 4.6–10.8) for *S*. *aureus*. Carriage prevalence by region is shown in Fig 1. Carriage differed significantly by region for *S*. *pneumoniae* and *M*. *catarrhalis* (p < 0.001 and p = 0.015, respectively, chi-squared test). To evaluate whether differences in carriage prevalence were due to differences in risk factors among regions, adjusted odds ratios (aOR) were determined by logistic regression models that included income, the presence of two or more children under five in the household, maternal education level, and presence of upper respiratory tract infection (URTI) symptoms. The difference in carriage prevalence of *S*. *pneumoniae* and *M*. *catarrhalis* among regions remained significant. For *S*. *pneumoniae*, aOR compared to Padang (reference) was 3.47 (95%CI 1.87–6.53) for Bandung and 1.78 (95%CI 0.93–3.42) for Central Lombok (p = 0.0006, chi-squared test). For *M*. *catarrhalis*, aOR compared to Padang was 1.60 (95%CI 0.86–2.97) for Bandung and 2.60 (95%CI 1.36–4.97) for Central Lombok (p = 0.015, chi-squared test).

**Fig 2:**
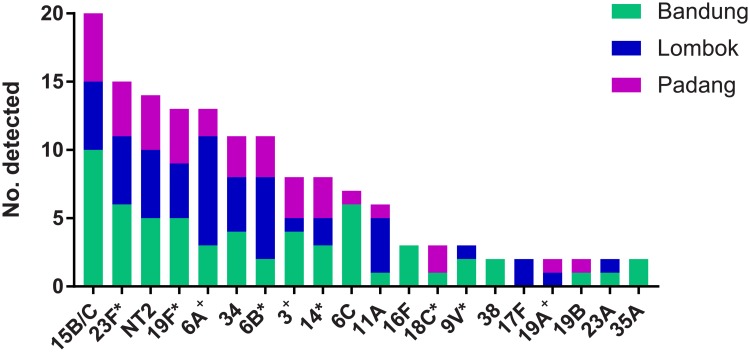
The twenty most common pneumococcal serotypes identified in nasopharyngeal swabs collected from Indonesian children aged 12–24 months, shown by region (Bandung, Padang, and Lombok). * indicates serotypes included in PCV10 and ^+^ indicates the additional three serotypes included in PCV13.

A total of 164 pneumococci belonging to 32 capsular serotypes and one genetic lineage of acapsular pneumococci (NT2) were identified in this study [25]. Five *lytA*-positive samples were culture negative and therefore not serotyped, and one sample was excluded for technical issues. There were four pneumococci for which serotype could not be determined: three were reported as 35A/10B-like by microarray (two of these were serogroup 33 by latex agglutination/Quellung whilst the third did not undergo phenotypic typing), and the fourth was 39/6C-like by microarray and non-typeable by latex. Two of the six serotype 11A pneumococci identified were typed as 11F-like by microarray, as previously described [26]. Multiple serotype carriage was found in 18/296 (6.1%) of samples overall, and in 18/144 (12.5%) of pneumococcal-positive samples. Serotypes 15B/C, 23F, NT2, 19F, and 6A were the most commonly identified. Fig 2 depicts the 20 most common serotypes shown by region. Overall, 76/164 (46.3%) of pneumococci belonged to PCV13 serotypes, and 72/296 (24.3%) of participants carried a PCV13 serotype. For PCV10, these proportions were 53/164 (32.3%) and 52/296 (17.6%), respectively. Serotype distribution varied significantly by region, with 26/73 (36%) of pneumococci in Bandung, 28/53 (53%) in Central Lombok, and 22/38 (58%) in Padang belonging to PCV13 serotypes (p = 0.043, chi-squared test). None of the 83 samples positive for *H*. *influenzae* were type B; further typing of *H*. *influenzae* was not conducted.

Relationships between colonizing species were examined by calculating odds ratios (OR) and aOR adjusting for income, the presence of two or more children under five in the household, maternal education level, and presence of URTI symptoms to minimize potential confounding. A positive association was observed between carriage of *S*. *pneumoniae* and *M*. *catarrhalis* (OR 3.07 [95%CI 1.91–4.94], aOR 2.85 [95%CI 1.72–4.72]) and *H*. *influenzae* and *M*. *catarrhalis* (OR 2.34 [95%CI 1.40–3.91], aOR 2.18 [95%CI 1.28–3.72]), and a negative association was found between *M*. *catarrhalis* and *S*. *aureus* (OR 0.06 [95%CI 0.01–0.43], aOR 0.06 [95%CI 0.01–0.50]). No significant associations were observed between *S*. *pneumoniae* and *H*. *influenzae* (OR 1.58 [95%CI 0.95–2.65], aOR 1.64 [95%CI 0.95–2.83]), *S*. *pneumoniae* and *S*. *aureus* (OR 0.49 [95%CI 0.19–1.24], aOR 0.58 [95%CI 0.22–1.52]), or *H*. *influenzae* and *S*. *aureus* (OR 0.25 [95%CI 0.06–1.07], aOR 0.27 [95%CI 0.06–1.21]).

**Fig 3:**
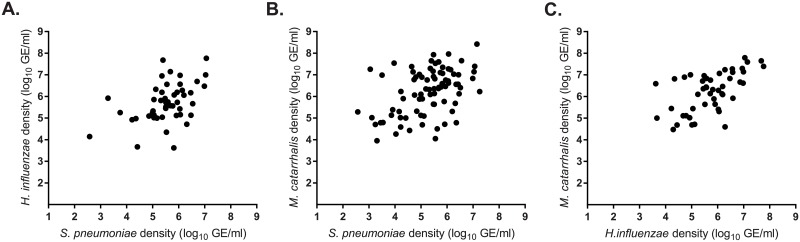
Density of *S*. *pneumoniae* and *H*. *influenzae* (A), *S*. *pneumoniae* and *M*. *catarrhalis* (B), and *M*. *catarrhalis* and *H*. *influenzae* (C) when present as co-colonizing species. Density are reported in log_10_ genome equivalents/ml.

In children positive for carriage, the median density of carriage was 5.1 log_10_ GE/ml (range 2.2–7.3) for *S*. *pneumoniae*, 5.6 log_10_ GE/ml (range 3.6–7.8) for *H*. *influenzae*, 6.2 log_10_ GE/ml (range 3.9–8.5) for *M*. *catarrhalis*, and 4.3 log_10_ GE/ml (range 3.6–7.0) for *S*. *aureus*. As seen in Table 2, *S*. *pneumoniae* densities were higher when co-colonizing with *H*. *influenzae* or *M*. *catarrhalis*. Similarly, *H*. *influenzae* density was higher when it co-occurred with *M*. *catarrhalis*, and *M*. *catarrhalis* density was significantly higher when *S*. *pneumoniae* was a co-colonizer. During co-colonization, densities of *S*. *pneumoniae*, *H*. *influenzae*, and *M*. *catarrhalis* were positively correlated (Fig 3): *S*. *pneumoniae* and *H*. *influenzae* Spearman ρ = 0.430, p = 0.002; *S*. *pneumoniae* and *M*. *catarrhalis* Spearman ρ = 0.400, p = 0.0002; *H*. *influenzae* and *M*. *catarrhalis* Spearman ρ = 0.571, p <0.0001. An opposite trend was observed for *S*. *pneumoniae* and *S*. *aureus*, as the median carriage density of *S*. *pneumoniae* was 4.61 log_10_ GE/ml with *S*. *aureus* compared to 5.18 log_10_ GE/ml without *S*. *aureus*, although the difference was not statistically significant (p = 0.09, Mann-Whitney test).

**Table 2:** Median density in log_10_ genome equivalents/ml of pneumococcus (SP), *H*. *influenzae* (HI), and *M*. *catarrhalis* (MC) when found with (+) or without (-) another species.

Colonizing species	+ HI	- HI	P value^a^	+ MC	- MC	P value	+ SP	- SP	P value
*S*. *pneumoniae*	5.54	4.79	<0.0001	5.48	4.78	0.0017			
*H*. *influenzae*				5.77	5.15	0.02	5.69	5.15	0.058
*M*. *catarrhalis*	6.39	6.10	0.367				6.41	5.86	0.048

^a^Mann-Whitney test

## Discussion

We examined nasopharyngeal carriage of four clinically-relevant bacterial pathogens in Indonesian children living in three diverse regions. The carriage prevalence of *H*. *influenzae* and *S*. *aureus* were similar among the three regions, whereas carriage of *S*. *pneumoniae* and *M*. *catarrhalis* varied. The reasons for regional differences are unclear. Samples were collected during the same time frame, limiting seasonal effects, and differences remained after adjusting for risk factors related to socioeconomic status, exposure, and presence of URTI symptoms. Regional differences in carriage may relate to other risk factors not assessed in this study, ethnic differences, or environmental differences. The overall pneumococcal carriage prevalence in this study (49.5%) is consistent with previous carriage studies of children under five conducted in Lombok (46%) and Semarang, Java (43%) [12, 14]. The 27.5% carriage rate of *H*. *influenzae* is similar to the 32% prevalence previously reported in Lombok in children aged 0–24 months [15]. In the 1998 Lombok study, carriage prevalence of Hib was 4.5%, whereas no Hib was detected in the current study, likely due to widespread use of Hib vaccine following its introduction in 2013. Carriage prevalence of *S*. *aureus* was low (7.3%), however this is expected as *S*. *aureus* carriage is highest in neonates, older children and adults, and low in children aged 12–24 months [8, 9].

Overall, 46% of pneumococci belonged to PCV13 serotypes. Due to limited serotyping data available from invasive pneumococcal disease in Indonesia, monitoring serotype changes in pneumococcal carriage may be a practical way of assessing vaccine impact. The distribution of pneumococcal serotypes varied among regions, ranging from 58% of serotypes carried by children in Padang belonging to PCV13 serotypes compared to 36% for children in Bandung. This finding has implications for the upcoming introduction of PCV13, as the proportion of circulating pneumococci with serotypes covered by the vaccine may vary among regions. Carriage of nonencaspulated pneumococci was common, consistent with reports from Nepal and Thailand [27, 28]. The genetic variants identified by microarray (36A/10B-like and 39/6C-like) warrant further investigation, as they could represent novel pneumococcal variants or potential new serotypes.

In this study, associations between *S*. *pneumoniae*, *H*. *influenzae*, and *M*. *catarrhalis* were consistently positive. In contrast, *S*. *aureus* carriage was negatively associated with *M*. *catarrhalis*, and trended towards negative associations with *S*. *pneumoniae* and *H*. *influenzae*. These observations are consistent with the literature [7]. Densities of *S*. *pneumoniae* and *H*. *influenzae* were positively associated in studies conducted in healthy children in Peru and Israel, similar to our results [10, 11].

A strength of the current study is the contemporaneous collection of samples from children living in distinct regions of Indonesia, which enabled the comparison of carriage prevalence among regions, and acquisition of the first data on carriage of *M*. *catarrhalis* and *S*. *aureus* in healthy children in this populous country. Although qPCR was used for detection of all four pathogens examined, confirmation of equivocal results (Ct values between 35–40) by culture was only conducted for *S*. *pneumoniae*, therefore the limit of detection was higher for *M*. *catarrhalis*, *S*. *aureus*, and *H*. *influenzae*, and these species may not have been detected if present at low densities. Another limitation of the study is that respiratory viruses, an important component of the microbial community of the upper respiratory tract, were not assessed [29].

Bacterial density, and presence of multiple pathogens, has been linked to disease in several studies, however the mechanisms underlying these interactions are not well understood. In a study on children under 10 years of age in Tanzania [30], density of *S*. *pneumoniae*, *H*. *influenzae*, and *M*. *catarrhalis* was significantly higher in children with severe pneumonia compared to those with mild UTRI, and carriage of multiple species was more common in children with clinical pneumonia compared to those without respiratory infection. In Vietnamese children under five, nasopharyngeal density of pneumococcus was higher in children with radiologically confirmed pneumonia compared to healthy controls or children with other lower respiratory tract infection [5]. In both the Tanzanian and Vietnamese studies, nasopharyngeal sampling was conducted during acute illness and prior to antibiotic treatment. Co-colonization of *S*. *pneumoniae* and *H*. *influenzae* was significantly associated with radiologically confirmed pneumonia, whereas co-colonization of *H*. *influenzae* and *M*. *catarrhalis* was associated with other lower respiratory tract infection. *S*. *pneumoniae*, *H*. *influenzae*, and *M*. *catarrhalis* are the three most common bacterial causes of otitis media, which is increasingly recognized as a polymicrobial disease [31]. Co-colonization of *S*. *pneumoniae* or *H*. *influenzae* with *M*. *catarrhalis* has been associated with increased risk of otitis media [32]. Using *in vivo* models, mixed species biofilms have been found to increase persistence in ear disease [33]. Other proposed mechanisms for positive associations between bacterial species include interspecies quorum sensing and passive antimicrobial resistance, which have been observed in experimental models of otitis media [34, 35]. Host susceptibility may also be an underlying factor for positive associations observed between certain bacterial species. For example, a study in Fiji found that that co-colonization by multiple pathogens was associated with ethnicity [36].

Continued surveillance of pneumococcal carriage and serotype distribution is warranted in Indonesia, especially in light of the upcoming introduction of pneumococcal conjugate vaccine, as our results suggest that vaccine impact may vary by region. Future studies on microbial interactions in the respiratory tract, ideally including viruses as well as bacterial pathogens, will help to shed light on the underlying mechanisms, as well as how nasopharyngeal microbiology relates to the development of respiratory infections such as pneumonia and otitis media.
